# High Fat Diet Accelerates Pathogenesis of Murine Crohn’s Disease-Like Ileitis Independently of Obesity

**DOI:** 10.1371/journal.pone.0071661

**Published:** 2013-08-16

**Authors:** Lisa Gruber, Sigrid Kisling, Pia Lichti, François-Pierre Martin, Stephanie May, Martin Klingenspor, Martina Lichtenegger, Michael Rychlik, Dirk Haller

**Affiliations:** 1 Chair of Nutrition and Immunology, Technische Universität München, Freising-Weihenstephan, Germany; 2 Biofunctionality Unit, ZIEL - Research Center for Nutrition and Food Sciences, Technische Universität München, Freising-Weihenstephan, Germany; 3 Nestec Ltd., Nestlé Research Center, Lausanne, Switzerland; 4 Nestlé Institute of Health Sciences SA, Campus EPFL, Lausanne, Switzerland; 5 Nutritional Medicine Unit, ZIEL - Research Center for Nutrition and Food Sciences, Technische Universität München, Freising-Weihenstephan, Germany; 6 Chair of Molecular Nutritional Medicine, Technische Universität München, EKFZ - Else Kröner-Fresenius-Center for Nutritional Medicine, Freising-Weihenstephan, Germany; 7 Chair of Analytical Food Chemistry, Technische Universität München, Freising-Weihenstephan, Germany; 8 BIOANALYTIK Weihenstephan, ZIEL - Research Center for Nutrition and Food Sciences, Technische Universität München, Freising-Weihenstephan, Germany; Charité-University Medicine Berlin, Germany

## Abstract

**Background:**

Obesity has been associated with a more severe disease course in inflammatory bowel disease (IBD) and epidemiological data identified dietary fats but not obesity as risk factors for the development of IBD. Crohn’s disease is one of the two major IBD phenotypes and mostly affects the terminal ileum. Despite recent observations that high fat diets (HFD) impair intestinal barrier functions and drive pathobiont selection relevant for chronic inflammation in the colon, mechanisms of high fat diets in the pathogenesis of Crohn’s disease are not known. The aim of this study was to characterize the effect of HFD on the development of chronic ileal inflammation in a murine model of Crohn’s disease-like ileitis.

**Methods:**

TNF^ΔARE/WT^ mice and wildtype C57BL/6 littermates were fed a HFD compared to control diet for different durations. Intestinal pathology and metabolic parameters (glucose tolerance, mesenteric tissue characteristics) were assessed. Intestinal barrier integrity was characterized at different levels including polyethylene glycol (PEG) translocation, endotoxin in portal vein plasma and cellular markers of barrier function. Inflammatory activation of epithelial cells as well as immune cell infiltration into ileal tissue were determined and related to luminal factors.

**Results:**

HFD aggravated ileal inflammation but did not induce significant overweight or typical metabolic disorders in TNF^ΔARE/WT^. Expression of the tight junction protein Occludin was markedly reduced in the ileal epithelium of HFD mice independently of inflammation, and translocation of endotoxin was increased. Epithelial cells showed enhanced expression of inflammation-related activation markers, along with enhanced luminal factors-driven recruitment of dendritic cells and Th17-biased lymphocyte infiltration into the lamina propria.

**Conclusions:**

HFD feeding, independently of obesity, accelerated disease onset of small intestinal inflammation in Crohn’s disease-relevant mouse model through mechanisms that involve increased intestinal permeability and altered luminal factors, leading to enhanced dendritic cell recruitment and promoted Th17 immune responses.

## Introduction

Inflammatory bowel diseases (IBD) such as Crohn’s disease (CD) and ulcerative colitis (UC) are spontaneously relapsing immunologically mediated disorders of the gastrointestinal tract. Despite extensive research, environmental factors relevant for the development of IBD are largely unknown. According to the current paradigm a combination of a given host genetic susceptibility, a certain microbial aggressiveness and possibly other environmental triggers leads to inappropriate host immune response toward an otherwise non-pathogenic microbiota. Parallel to the progression of industrialization and coinciding with a high prevalence of obesity and associated metabolic disorders, the incidence of IBD has risen considerably over the last decades. Although IBD patients have historically been lean and even malnourished due to inflammation-associated malabsorption, recent studies report increasing rates of overweight IBD patients [1], [2], and associate obesity with a more severe disease course [1], [3].

A recent systematic literature review of epidemiological data from 19 studies comprising 1,269 CD and 1,340 UC patients concluded that a high dietary intake of fat, adjusted for total energy intake and behavioral confounders, increases the risk of IBD development [4]. In addition, animal studies indicate that high-fat diet (HFD)-induced obesity promotes intestinal inflammatory processes on a cellular level [5], and perturbs barrier function [6], but the underlying mechanisms remain largely unidentified. Whether the observed effects are attributed to HFD or obesity as a (patho-)physiological condition is under vivid debate [7]–[9]. It is therefore crucial to identify whether a systemic inflammatory metabolic tone in the host or the impact of the diet on luminal factors are the essential triggers for the promotion of pathogenesis. CD is characterized by a discontinuous and often granulomatous chronic inflammatory phenotype of the entire digestive tract. Typical histologic features of this T helper cell (Th) 1/Th17-driven transmural immunopathology largely affect the terminal ileum (50–70%) and to some extent the colon [10], [11]. Despite recent evidence for the impact of HFD on the selection of a disease-relevant microbiota in an IBD-related mouse model for chronic inflammation in the colon, the role of HFD in the pathogenesis of chronic inflammation in the small intestine are not known.

In the present study we investigated the effect of HFD on the pathogenesis in TNF^ΔARE/WT^ mice, a genetic mouse model of Crohn’s disease-like ileitis, and its relation to changes in metabolic phenotype, intestinal permeability and immune response. Heterozygous TNF^ΔARE/WT^ mice spontaneously develop T-cell-dependent ileitis closely resembling the immune and tissue-related phenotype of human Crohn’s disease with ileal involvement [12]. Intestinal inflammation in the TNF^ΔARE/WT^ mice is associated with early reduction of CD8αα-expressing intraepithelial lymphocytes with concomitant predominance of TNF-producing CD8αβ T-cells in the epithelial layer, as well as decreased Th1 and increased Th17 responses by CD4^+^ lymphocyte [12]. We reported previously that TNF^ΔARE/WT^ mice develop progressing ileitis from 8 weeks of age onwards in our experimental surrounding, with marked leukocyte infiltration and architectural disruption. In addition, this animal model shows diminished body weight and adipose tissue mass compared to matched C57BL/6 wildtype (WT) mice, despite comparable energy uptake [13].

We challenged this mouse model with a 48 kJ% HFD and investigated the metabolic phenotype as well as mechanisms of intestinal inflammation.

## Materials and Methods

### Animals and Experimental Protocol

Heterozygous C57BL/6 TNF^ΔARE/WT^ mice and WT littermates as well as SWR/J mice were conventionally raised at constant room temperature (22±2°C), air humidity (55±5%), and a light/dark cycle of 12/12 h. Water and diets (ssniff, Soest, Germany) were provided ad libitum. At 4 weeks of age, animals were divided into feeding groups and received either control diet (12 kJ% fat) or palm oil-based high-fat diet (HFD; 48 kJ% fat) until the ages of 8, 12 or 16 weeks. Detailed information on the diet composition is provided in Table S1, including the re-analyzed fatty acid profile of the diets. Disease Activity Index (DAI) was assessed weekly as a combined score of weight loss, stool consistency and rectal bleeding, adapted from Cooper et al. [14]. EDTA-blood was collected from the *vena cava inferior* and the *vena portae hepatis*, centrifuged at 3000 g for 10 min and plasma stored at −80°C until analysis. Tissue samples were removed and stored immediately at −80°C, or fixed in 4% formalin for histological processing.

### Ethics Statement

Animal experiments were conducted according to relevant national and international guidelines. All performed animal experiments were approved by the Bavarian Animal Care and Use Committee under the application number AZ 55.2-1-54-2531-130-09.

### Glucose Tolerance Test

Oral glucose tolerance tests were performed as follows: 6-h-fasted mice were gavaged with a 20% glucose solution in 0.9% NaCl (2 g glucose/kg body weight). Blood glucose concentration was determined before and 15 min, 30 min, 60 min and 120 min after application with a glucose meter (FreeStyle Lite, Abbot Diabetes Care, Alameda, CA, USA) using one drop of blood collected from the tip of the tail. The area under the curve was calculated using the trapezoidal rule.

### Histological Scoring of Intestinal Tissue

Formalin-fixed intestinal samples were embedded in paraffin and cut in sections of 5 µm followed by hematoxylin and eosin (H&E) staining. Histological scoring was performed by blindly assessing the degree of lamina propria (LP) mononuclear cell infiltration, crypt hyperplasia, goblet cell depletion and architectural disruption in the different gut sections, resulting in a score from 0 (not inflamed) to 12 (highly inflamed), as previously described [15].

### Adipocyte Size Measurements

Formalin-fixed mesenteric adipose tissue (MAT) samples were embedded in paraffin and cut in sections of 5 µm followed by H&E staining. The mean adipocyte size was determined in stained fat sections at a magnification of 200-fold and the size of adipocytes was calculated using AxioVision 4.6 (Carl Zeiss AG, Jena, Germany).

### Isolation of Intestinal Epithelial Cells

Primary intestinal epithelial cells (IEC) from the ileal epithelium were purified as previously described [16]. Briefly, the fresh ileal tissue was opened longitudinally, washed in PBS and incubated at 37°C in DMEM containing 10% FCS, 1% Glutamine, 1% antibiotic-antimycotic (all Invitrogen, Carlsbad, CA, USA) and 1mM DTT for 20 min, followed by vortexing for 1 min. The remaining tissue was incubated in 30 ml PBS containing 1.5 mM EDTA for additional 20 min, followed by vortexing for 1 min. The supernatants were centrifuged for 5 min at 350 g and the cell pellets were re-suspended in DMEM containing 5% FCS. Finally, the primary IEC suspension was purified by centrifugation through a 20%/40% discontinuous Percoll gradient at 600 g for 30 min. Purified IECs were washed in PBS, lysed for further processing and stored at −80°C.

### Gene Expression Analysis

Total RNA was isolated from IEC, adipose tissue, isolated lymphocytes or bone-marrow derived dendritic cells using a phenol-based extraction (Qiagen, Hilden, Germany). RNA yield and quality were assessed by absorbance using a Nanodrop ND-1000 spectrophotometer (LabTech International, Brampton, ON, Canada). A total of 1 µg RNA was used for reverse transcription using M-MLV point mutant system (Promega, Fitchburg, WI, USA). Primers and probes were designed using the universal probe library (Roche, Mannheim, Germany). RNA-expression profiles were analyzed using the LightCycler (Roche) with 400 nM primers, 200 nM probe and 1 µl cDNA (QuantiTect Probe RT-PCR Master Mix buffer) in a quantitative real-time PCR (qPCR).The relative induction of mRNA expression was calculated using the equation 2^−ΔΔCp^ and normalized for the expression of *gapdh.* Data were expressed as -fold change against WT on control diet.

### Cytokine and Chemokine Quantification

Plasma specimen or cell culture supernatants were used to quantify CCL20 using Quantikine ELISA kit (R&D Systems Europe, Abingdon, UK). Plasma Leptin, IL6, TNF and MCP1 were quantified in plasma using a bioplex system (BioRad, München, Germany).

### Metabonomic Analyses of Plasma and Ileal Tissues

For targeted LC-MS analysis of intestinal tissue, a sample piece of the distal ileum was excised and flushed with 1 ml phosphate buffered saline using a sterile syringe and stored at −80°C. Metabolites were measured from homogenized ileal tissue and plasma using the Biocrates Life Sciences AbsoluteIDQ kit. For further details on the analyses please see Supporting Information (Materials and Methods S1).

### Western Blot Analysis

Purified primary IEC of 12-week-old mice were lysed, homogenized using ultrasound and protein concentration was quantitated by Bradford method (Carl Roth, Karlsruhe, Germany). Protein lysates were boiled in Laemmli buffer at 95°C for 10 min. Equal amounts of proteins were resolved on 7–20% SDS-polyacrylamide gels and transferred by electroblotting to a nitrocellulose membrane. Anti-Occludin (Invitrogen), anti-E-Cadherin (Abcam), anti-CCL20/MIP-3α (Santa Cruz Biotechnology, Santa Cruz, CA, USA) and anti-β-Actin (MP Biomedicals, Illkirch, France) antibodies were used to detect immunoreactive proteins of interest, using an enhanced chemiluminescence light-detecting kit (Amersham, Arlington Heights, IL, USA).

### Immunefluorescence Staining

Formalin-fixed samples of 12-week-old mice were embedded in paraffin and cut in 5 µm sections, cooked in citrate buffer and stained with anti-Occludin (Invitrogen) and anti-E-Cadherin (Abcam, Cambridge, UK) to localize immunoreactive proteins of interest. Microscopic pictures were acquired at a magnification of 200- to 600-fold using the confocal microscope FluoView FV10i (Olympus, Hamburg, Germany) and processed using Volocity 3D Image Analysis Software (Perkin Elmer, Rodgau, Germany).

### Endotoxin Quantification in Plasma

Endotoxin levels in hepatic portal vein plasma of 12-week-old mice were quantified using a limulus amoebocyte lysate chromogenic endpoint assay (Hycult biotech, Uden, Netherlands). The assay protocol was optimized in order to yield acceptable recovery rates. Briefly, plasma was diluted 1∶10 in endotoxin-free water, heated to 70°C for 10 min, and measured with addition of Pyrosperse (Lonza, Walkersville, MD, USA).

### Isolation of Lamina Propria Lymphocytes and Flow Cytometry

Lamina propria lymphocytes (LPLs) from 12-week-old mice were isolated by 1h collagenase digestion (1 mg/ml in PBS with Ca^2+^ and Mg^2+^) of ileal tissue after removal of IEC as indicated above. The cells were filtered through a 70 µm cell strainer (Corning Incorporated, Corning, NY, USA), pre-treated with FcR block (Milteny Biotec, Bergisch Gladbach, Germany) and stained using anti-CD11c, anti-CD3e, anti-CD4 (all BD Biosciences, Franklin Lakes, NJ, USA) and anti-CD8α (AbD Serotec, Düsseldorf, Gemany). A total of 10,000 cells was acquired using a LSR II flow cytometer (BD Biosciences) and analyzed using BD FACSDiva software. Gating methods have been described elsewhere [17].

### Stimulation of Mode K Cells

The cell line Mode K was established by Vidal et al. via immortalization of murine small intestinal epithelial cells by SV40 large T gene transfer through a murine ecotropic virus[18]. The fact that the Mode K cell line is small intestinal, murine, non-adenocarcinoma and widely used in studies of small intestinal mucosal immunology and antigen-presentation [19] make it an appropriate cell line to be used in the context of the present work. The cell line (passage 15–20) was cultured in humidified 5% CO_2_ atmosphere at 37°C using 6well cell culture dishes in DMEM containing 10% FCS, 1% Glutamine and 1% antibiotic-antimycotic. At early confluency, cells were stimulated for 24 h with preparations of cecal content (50 µg protein/ml) or 20 ng/ml recombinant mouse TNF (Invitrogen). Mode K cell-conditioned media (CM) were harvested and stored at −80°C.

### Dendritic Cell Transmigration and Stimulation Assays

Dendritic cells (DCs) were generated from bone marrow of C57BL/6 mice (BM-DCs). Briefly, femurs and tibiae were removed, cleaned thoroughly, cut open on both sides under sterile conditions and flushed with PBS to collect bone marrow. After lysis of red blood cells, remaining cells were cultivated in DMEM with 10% FCS, 1% Pen/Strep, 2 mM β-mercaptoethanol, 15 ng/ml IL4 and 15 ng/ml GM-CSF (both PeproTech, London, UK) for 7 days. Suspension cells were phenotyped before performing migration assays, using anti-CD11c, anti-CD11b, anti-MHCII and anti-CD86 (all BD Biosciences) as described above. For migration assays, BM-DCs were used at a density of 5 *10?4 cells/ml in a transwell permeable support system with 3 µm pore size (Corning Incorporated), applying different Mode K CM as chemoattractants (see above) for 1 h. The potential of conditioned media to result in migration of BM-DCs through the membrane was normalized to spontaneous migration (chemotactic index). Data are representative for three independent experiments. For stimulation assays, BM-DCs were used at a density of 2.5 *10?5 cells/ml and stimulated with the different Mode K CM for 24 h. BM-DCs were then lysed and RNA was isolated for subsequent qPCR analysis as described above.

### Statistical Analysis

Statistical tests were performed using two-way ANOVA followed by the Holm-Sidak test to compare dietary groups within the same genotype and age, or unpaired Student’s t-test depending on the comparison of interest. Differences were considered significant if p-values were <0.05(*), <0.01(**) or <0.001(***). Superscript letters refer to p-values <0.05. Data are shown as mean ± SD or in the case of non-normally distributed data as median with lower and upper quartile (box-whisker-plot), unless stated otherwise.

Please see Supporting Information (Materials and Methods S1) for methods regarding analysis of energy content in feces, fatty acids in feed, polyethylene glycol permeability assay and cecal content preparations,.

## Results

### HFD Accelerates the Development of Intestinal Inflammation in TNF^ΔARE/WT^ Mice

**Figure 1:**
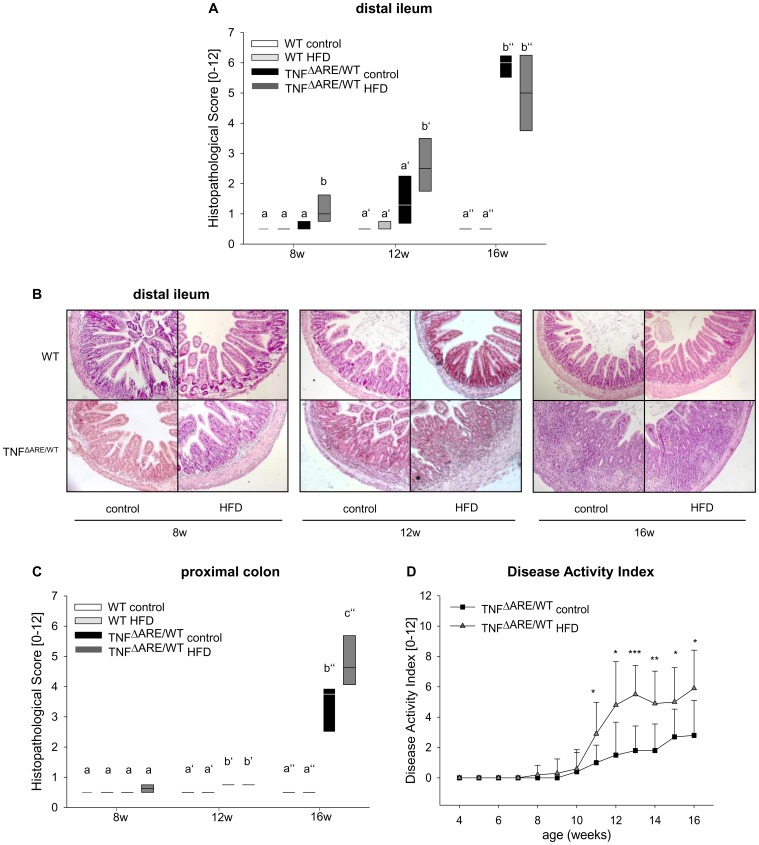
HFD accelerates the development of intestinal inflammation. WT and TNF^ΔARE/WT^ mice were fed control diet or HFD from the age of 4 weeks until the age of 8, 12 or 16 weeks. H&E stained sections of distal ileum (A, B) and proximal colon (C) were blindly assessed for the degree of histopathology. Data sets with different superscript letters differ significantly from each other. Disease activity index (DAI) was assessed as a combined score of weight loss, stool consistency and blood in stool (D). n = 6 per group for 8 weeks and 12 weeks, n = 9 per group for 16 weeks timepoint; Data sets with different superscript letters differ significantly from each other according to two-way ANOVA: a,b within 8 weeks, a’, b’ within 12 weeks, a”, b” within 16 weeks timepoint. *p<0.05 **p<0.01 ***p<0.001 between dietary groups within the same genotype according to Student’s t-test.

WT mice and TNF^ΔARE/WT^ mice were challenged at 4 weeks of age with 48 kJ% HFD and 12 kJ% control diet for different durations. At 8 weeks of age, ileal tissue pathology was significantly more severe in HFD compared to control diet, with further aggravation at 12 weeks of age (Figure 1A and Figure 1B). At 16 weeks of age the ileal histopathology level reached a plateau and did not differ between HFD and control mice (Figure 1A and Figure 1B). However, the secondarily affected proximal colon was more severely inflamed under HFD at the age of 16 weeks (Figure 1C). DAI was significantly higher in HFD animals from week 11 on (Figure 1D), underlining the finding of aggravated histopathology. Moreover, elevated fecal energy content in TNF^ΔARE/WT^ mice under HFD compared to control diet (18.23±1.63 MJ/kg vs. 12.96±0.52 MJ/kg) suggested that TNF^ΔARE/WT^ mice developed steatorrhea. WT mice showed no signs of lipid malabsorption and did not develop histopathological signs of inflammation along the intestine (Figure 1A-D).

### TNF^ΔARE/WT^ Mice do not Develop Obesity, Impaired Glucose Tolerance or Adipose Tissue Inflammation Under HFD

**Figure 2:**
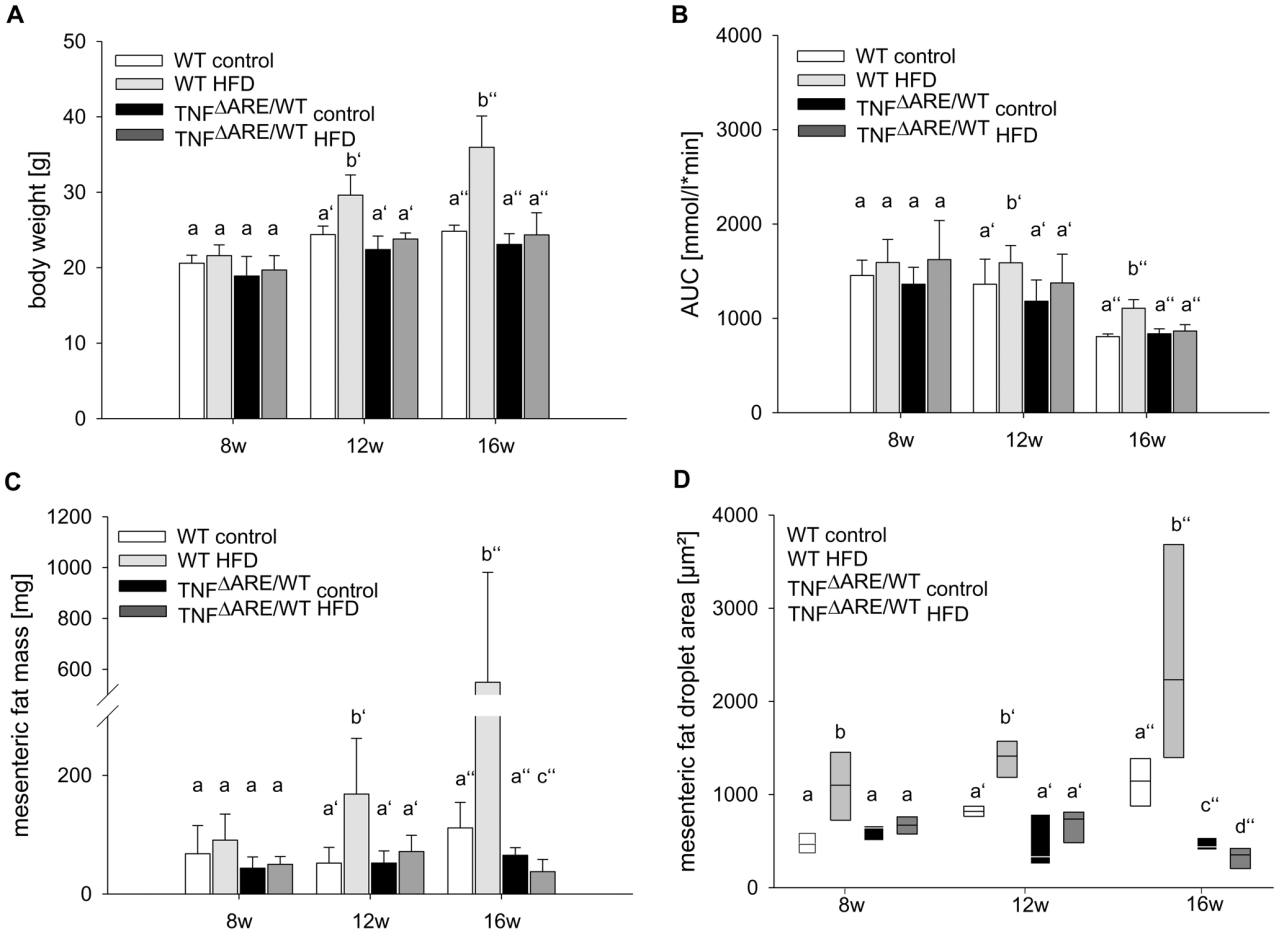
HFD fails to induce obesity or impaired glucose tolerance in TNF^ΔARE/WT^ mice. Body weight of WT and TNF^ΔARE/WT^ at the ages of 8, 12 and 16 weeks was assessed (A). Glucose tolerance was investigated *in vivo* by an oral glucose tolerance test (B). Tissue weights and (C) mean adipocyte size of mesenteric adipose depot (D) were determined. n = 6 per group for 8 weeks and 12 weeks, n = 9 per group for 16 weeks timepoint; Data sets with different superscript letters differ significantly from each other according to two-way ANOVA: a,b within 8 weeks, a’, b’ within 12 weeks, a”, b”; c”, d” within 16 weeks timepoint.

**Figure 3:**
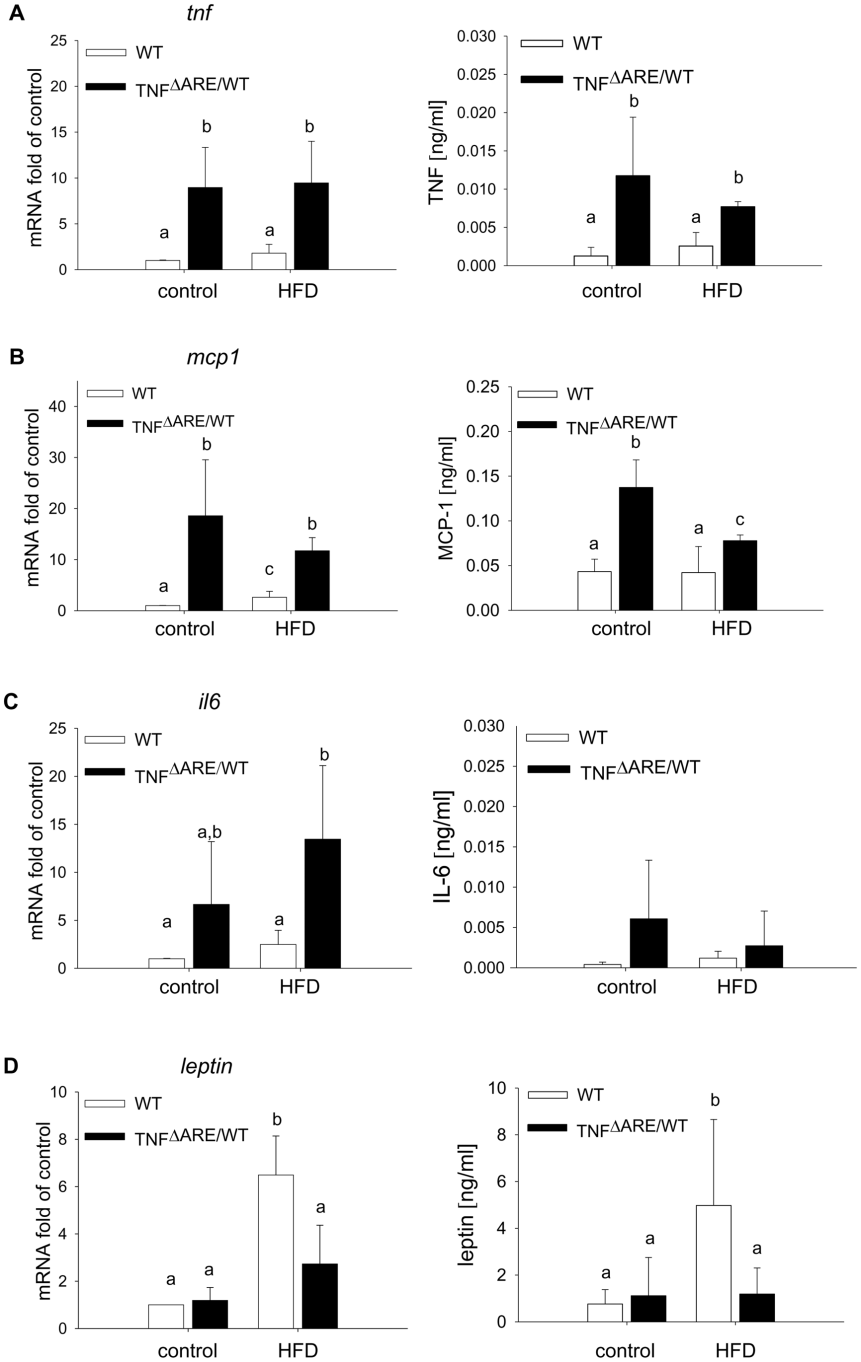
Lack of induction of inflammatory gene expression in adipose tissues of TNF^ΔARE/WT^ mice under HFD. Expression in adipose tissue and corresponding plasma levels of *tnf* (A), *mcp1* (B) and *il6* (C) and *leptin* (D) were analyzed in 12-week-old animals using qPCR and ELISA respectively. Cytokine/adipokine mRNA expression was normalized to *gapdh* expression and expressed as fold change relative to WT on control diet. n = 6 per group; data sets with different superscript letters differ significantly from each other according to two-way ANOVA.

**Figure 4:**
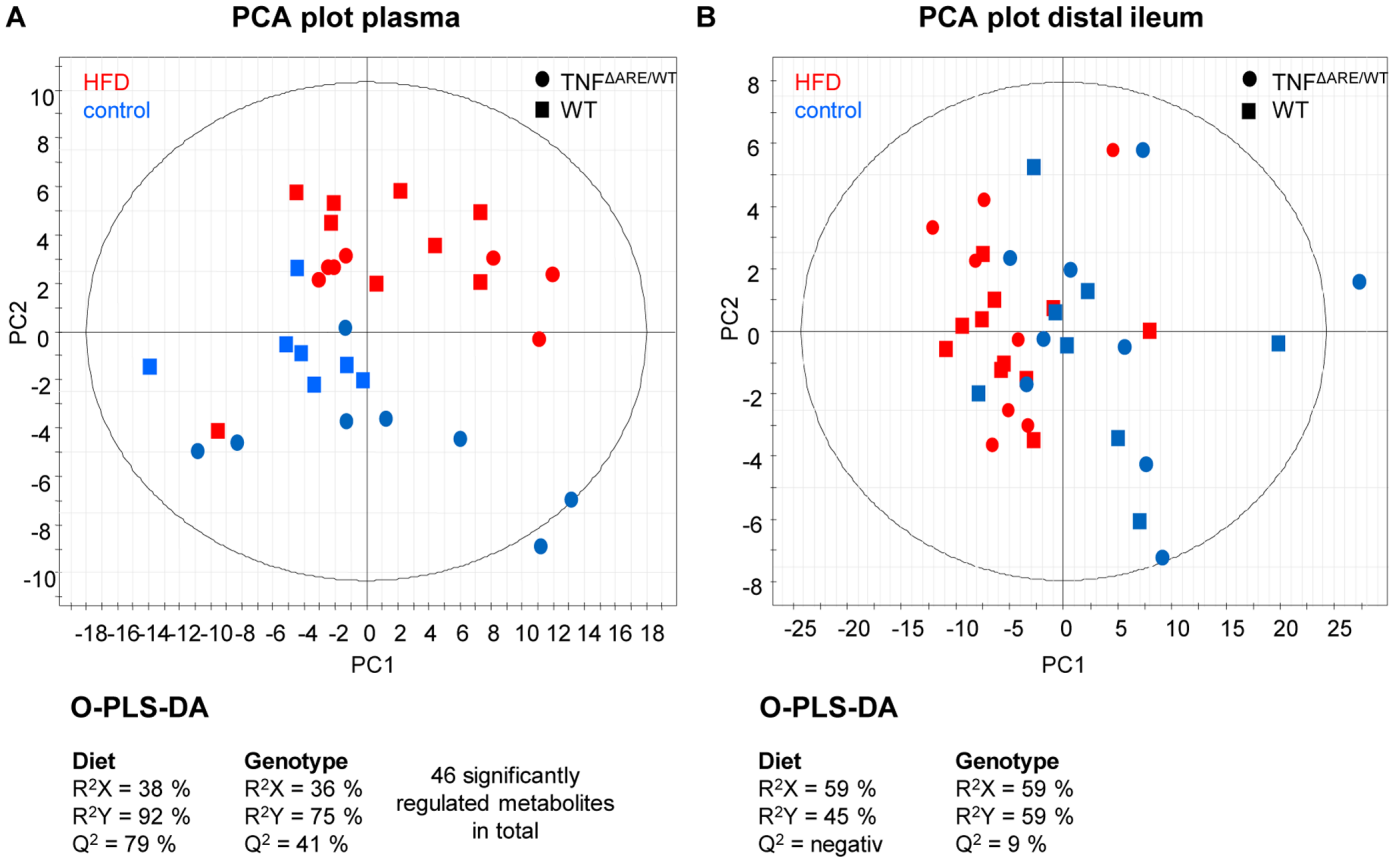
Metabolic composition of the plasma but not distal ileum is affected by the HFD. Metabolites in Plasma (A) and distal ileum tissue (B) of 12-week-old mice (n = 6 per group) were analyzed using the Biocrates Life Sciences AbsoluteIDQ kit. The metabolomics data are represented as PCA plots. Statistical analyses for dietary groups and genotype differences were then performed using O-PLS-DA.

To test the hypothesis that the aggravation of intestinal inflammation is associated with obesity and metabolic disorders, we investigated body weight development and glucose tolerance. In contrast to WT mice, TNF^ΔARE/WT^ mice did not develop obesity (Figure 2A) despite increased energy intake (Figure S1) over the course of the HFD feeding. At 12 and 16 weeks of age, fasting blood glucose was significantly elevated in WT mice under HFD and glucose tolerance was impaired, whereas glucose homeostasis remained normal in TNF^ΔARE/WT^ mice (Figure 2B). We next investigated the morphology of adipose tissue and local expression as well as systemic levels of adipokines typically associated with low-grade inflammation in obesity or HFD feeding. Again, in contrast to WT mice, TNF^ΔARE/WT^ mice did not show increased fat depots or enlarged adipocytes in MAT at any time point (Figure 2C and Figure 2D). At 12 weeks of age gene expression of TNF in MAT as well as TNF plasma levels were significantly up-regulated in TNF^ΔARE/WT^ compared to WT mice, with no further regulation in response to HFD (Figure 3A). MCP1 and IL6 gene expression were significantly up-regulated in MAT of TNF^ΔARE/WT^ mice without further regulation in response to HFD (Figure 3B and Figure 3C). The expression and plasma levels of leptin were strongly induced during HFD in WT mice, but not in TNF^ΔARE/WT^ mice (Figure 3D). Despite the unresponsiveness of the TNF^ΔARE/WT^ adipose tissue toward the HFD challenge, metabolic composition of the plasma reflected the dietary fatty acid profile independently of the genotype (Figure 4A). In particular, the HFD resulted in an upregulation of circulating levels of palmitoylcarnitine (C16), stearoylcarnitine (C18) and oleylcarnitine (C18∶1). Nevertheless, metabolic composition of the mainly affected tissue, the distal ileum, remained stable across genotypes and dietary groups (Figure 4B).

### Loss of Occludin Expression in the Distal Ileum and Endotoxin Translocation in Response to HFD

**Figure 5:**
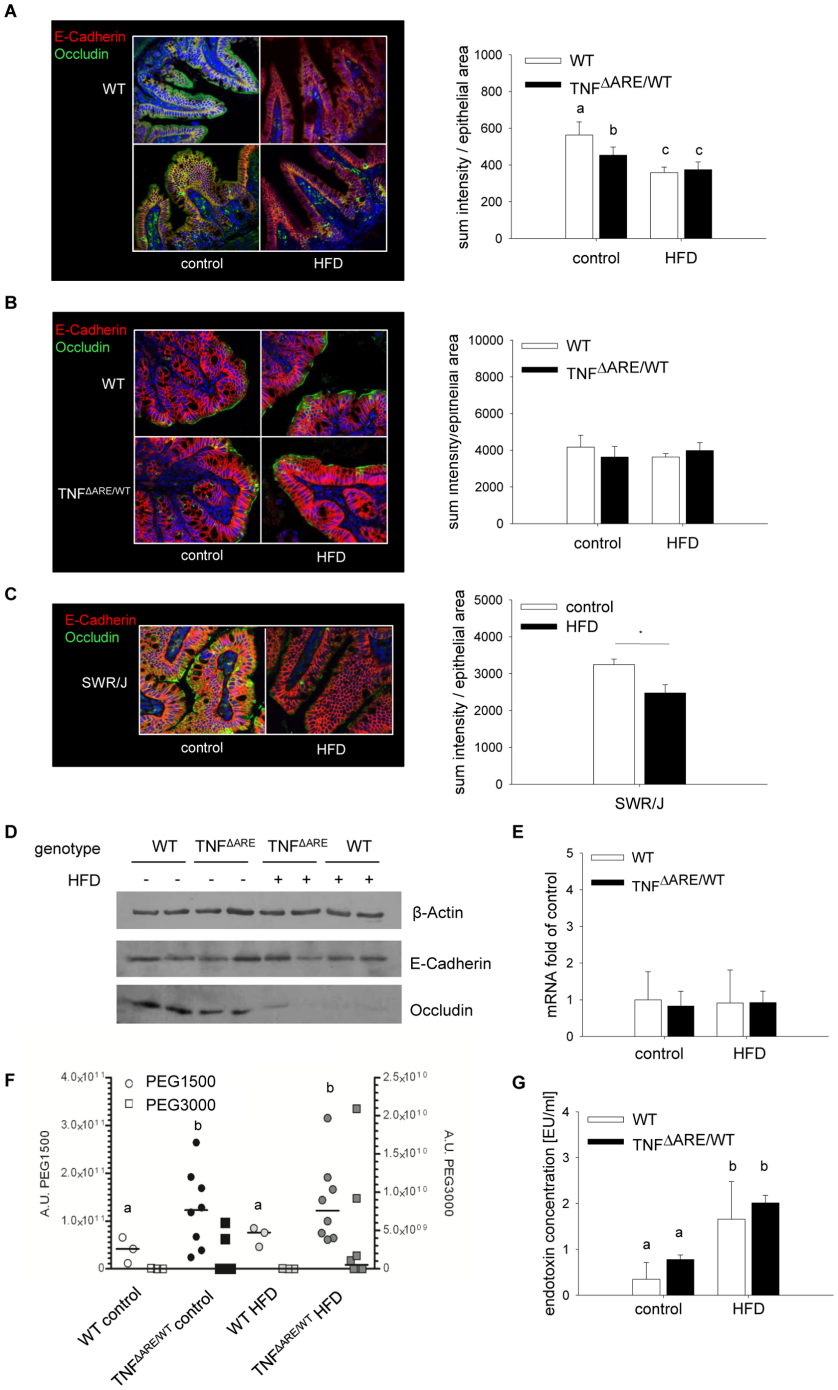
Loss of ileal occludin expression and increased endotoxin translocation but not PEG translocation upon HFD feeding. Protein expression of Occludin and E-Cadherin in ileal IEC was assessed by immunefluorescence analysis in distal ileum (A) and proximal colon (B) of 12-week-old TNF^ΔARE/WT^ mice and WT littermates (n = 4 per group), as well as ileum of SWR/J mice (C) (n = 4–5 per group) with subsequent quantification. Findings were confirmed by Western Blotting using β-actin as loading control (D). Gene expression of *occludin* was analyzed by qPCR (n = 6 per group) (E). Translocation of orally applied PEG of the average molecular sizes 1500 and 3000 Da were determined by LC/MS, determining the sum of sodium adducts as area units in counts per second in the respective SIM traces (F), and Plasma endotoxin concentrations in hepatic portal vein EDTA-plasma (G) were analyzed using an LAL Chromogenic Endpoint Assay (n = 6 per group). Data sets with different superscript letters differ significantly from each other according to two-way ANOVA; *p<0.05 according to Student’s t-test.

To investigate the effect of HFD on intestinal barrier integrity, we analyzed the expression of barrier proteins in the distal ileum as well as the translocation of bacterial components and of permeability markers *in vivo* and *ex vivo*. In the distal ileum protein expression of Occludin was dramatically reduced under HFD, independently of the mouse genotype (Figure 5A). E-Cadherin (Figure 5A) as well as other barrier proteins (data not shown) remained unchanged.

This selective loss of Occludin was found to be site-specific and did not occur in the proximal colon (Figure 5B). Furthermore, the dissociation of these HFD-associated effects from obesity was supported by the finding that SWR/J mice exhibited reduced ileal Occludin protein levels upon HFD in the same experimental setting (Figure 5C), despite their resistance toward HFD-induced obesity (Figure S1).

While we confirmed reduced protein expression in isolated IEC of TNF^ΔARE/WT^ mice and their WT littermates using Western blotting (Figure 5C), mRNA expression of *occludin* was unaltered throughout the groups (Figure 5D). The degree of PEG translocation was dependent on the molecular size (PEG 1500 vs PEG 3000) and the host genotype, but not on diet (Figure 5E). Parallel to the loss of Occludin in the ileal tissue but in contrast to the PEG translocation, plasma endotoxin levels in the portal vein were increased in HFD-fed mice independent of genotype (Figure 5F), suggesting that HFD increases LPS translocation.

### HFD Triggers Activation of Inflammatory Response in IEC

**Figure 6:**
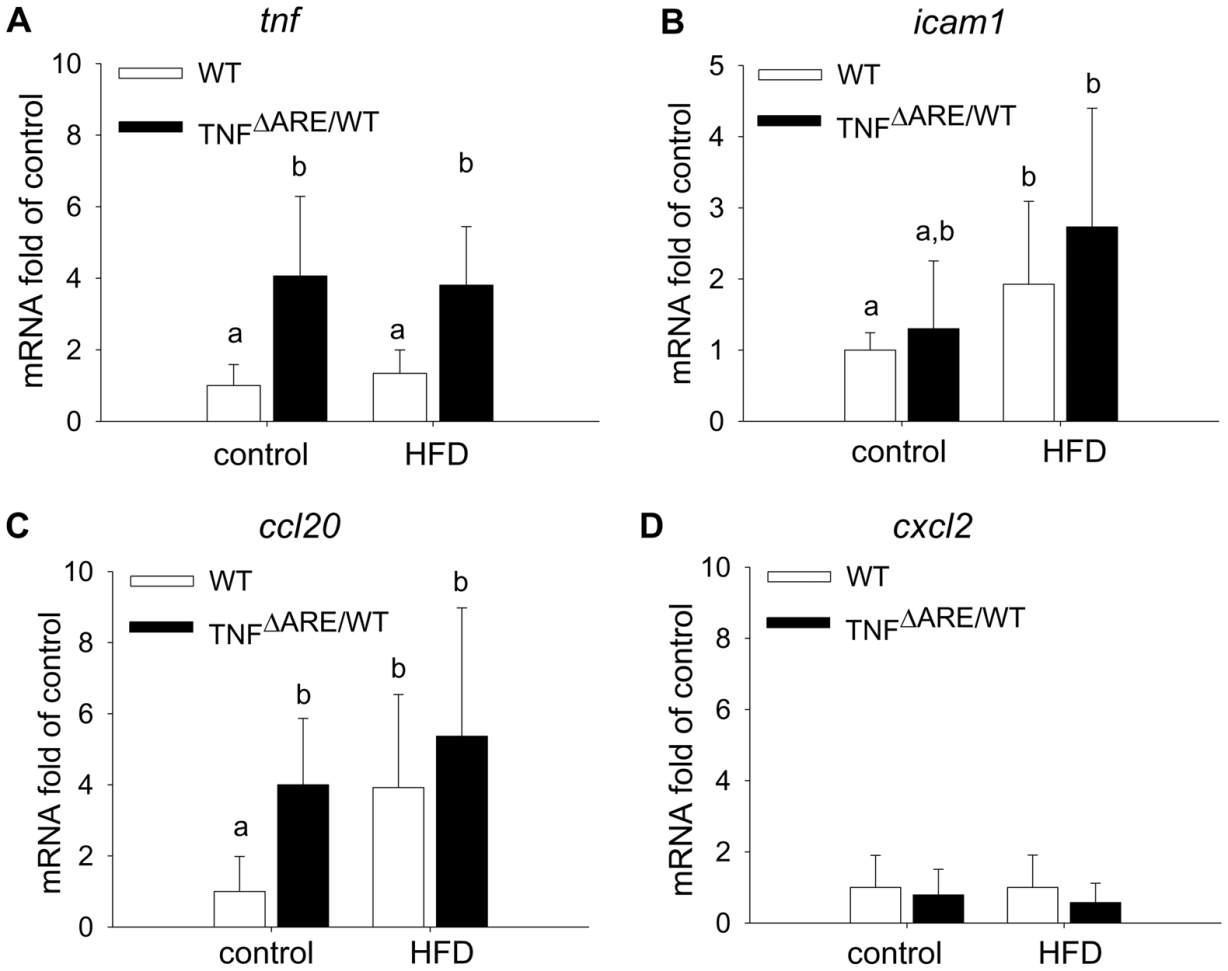
HFD triggers activation of inflammatory responses in IEC. Expression levels of *tnf* (A), *icam1* (B), *ccl20* (C) and *cxcl2* (D) mRNA were determined in isolated ileal IEC of 12-weeks-old animals using qPCR. Relative mRNA expression was normalized to *gapdh* and expressed as fold change relative to WT on control diet. n = 6 per group; Data sets with different superscript letters differ significantly from each other according to two-way ANOVA.

We were next interested in the molecular mechanisms underlying the aggravated inflammatory phenotype of HFD-fed TNF^ΔARE/WT^ mice. Since epithelial cell homeostasis is critical for the maintenance of a healthy gut, we investigated the effect of HFD on the expression of inflammation-associated activation markers in the ileal epithelium including cytokines, chemokines and cellular adhesion molecules. HFD did not modulate TNF expression in WT nor TNF^ΔARE/WT^ mice (Figure 6A). The expression of intercellular cell adhesion molecule 1 (*icam1*), functioning as a facilitator for lymphocyte transmigration, was up-regulated under HFD (Figure 6B). In addition, expression of the chemokine (C-C motif) ligand (*ccl*) *20*, targeting the recruitment of DCs, was induced by HFD in both genotypes (Figure 6C). Notwithstanding the regulation of *ccl20*, the expression of other chemokines such as chemokine (C-X-C motif) ligand (*cxcl*) *2,* which recruits primarily neutrophils, was not affected (Figure 6D).

### HFD Induces DC Recruitment to the Ileal LP is Associated to Luminal Intestinal Factors

**Figure 7:**
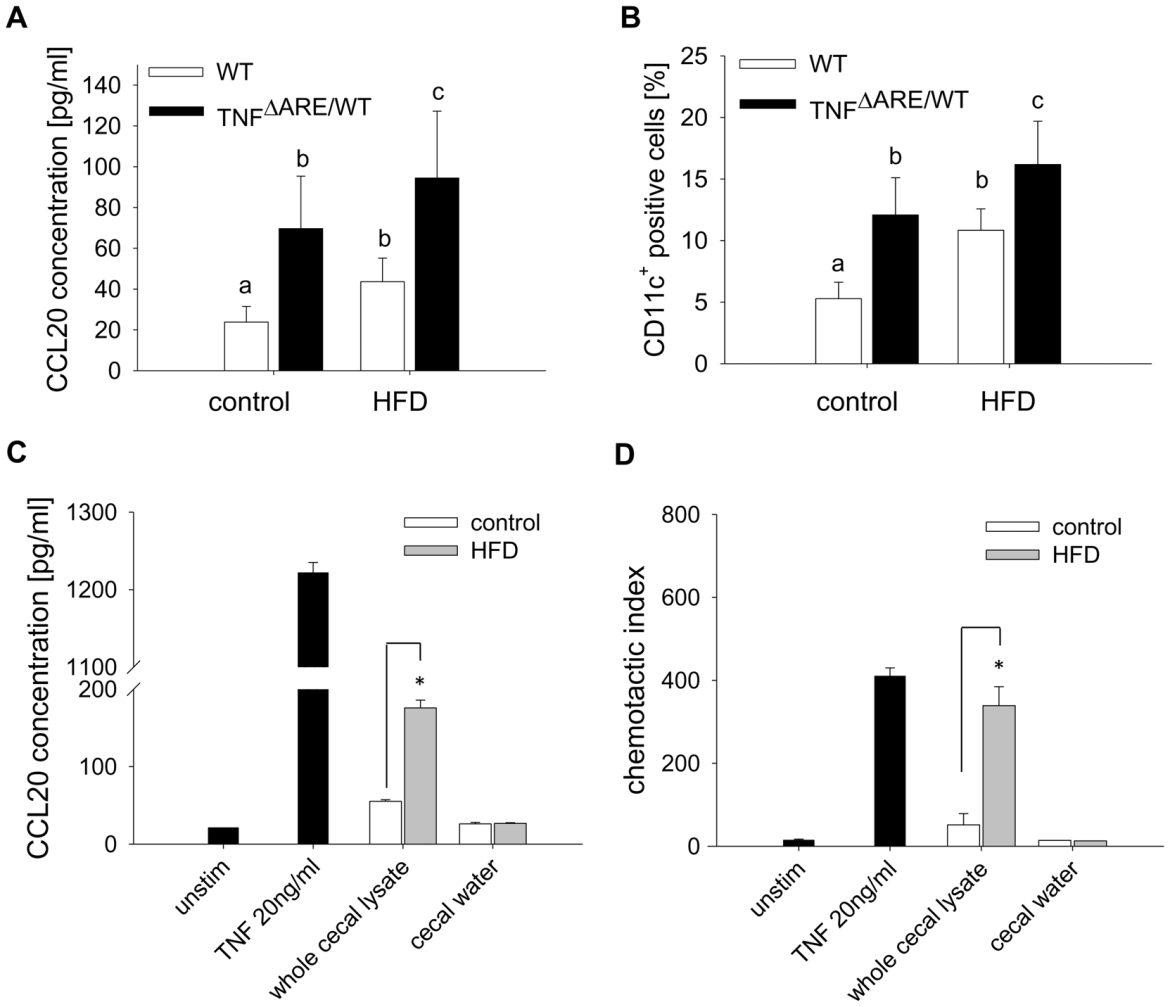
Cecal lysates trigger chemokine production and DC recruitment. Plasma CCL20 concentration was measured by ELISA (A), and CD11c^+^ dendritic cells in the LP were quantified by cell cytometry (B) (n = 6 per group). Mode K cells were stimulated in technical triplicates with cecal lysates (50 µg protein/ml) or cecal water (50 µg protein/ml) of mice fed the different diets for 24 h. TNF (20 ng/ml) was used as a positive control. CCL20 concentration was measured in the supernatants using ELISA (C). With these conditioned supernatants, the chemotaxis of DCs generated from bone-marrow was assessed in triplicates using a transwell migration assay (D). Data are representative for three independent experiments. Data sets with different superscript letters differ significantly from each other according to two-way ANOVA; *p<0.05 according to Student’s t-test.

Since IECs were found to express increased levels of *ccl20*, translating into elevated CCL20 plasma levels (Figure 7A), we next quantified CD11c^+^ DCs in the ileal LP using flow cytometry. Indeed, the number of CD11c^+^ cells in the ileal LP was significantly increased in response to HFD in both genotypes, with significant higher numbers in TNF^ΔARE/WT^ mice (Figure 7B). To investigate possible mechanisms *in vitro,* Mode K small intestinal epithelial cells were stimulated with lysates from the cecal content of mice fed the different diets. Mode K cells secreted significantly more CCL20 upon treatment with HFD-derived compared to control diet-derived cecal content (Figure 7C). In contrast, cecal water did not result in differential CCL20 production, suggesting that not metabolites but other HFD-associated luminal components drive this effect. CCL20 production upon treatment with suspensions of the pure diets was not increased under HFD conditions (1 µg/ml: 24.7±2.4 vs. 21.7±0.7; 50 µg/ml: 58.4±2.2 vs 43.4±2.9; for control and HFD respectively). According to the CCL20 production in stimulated cell culture experiments, the supernatants of Mode K cells stimulated with HFD-derived cecal lysate were much more potent to recruit BM-DCs compared to control diet-derived lysate (Figure 7D).

### HFD Drives Increased Th17 Polarization in TNF^ΔARE/WT^ Mice

**Figure 8:**
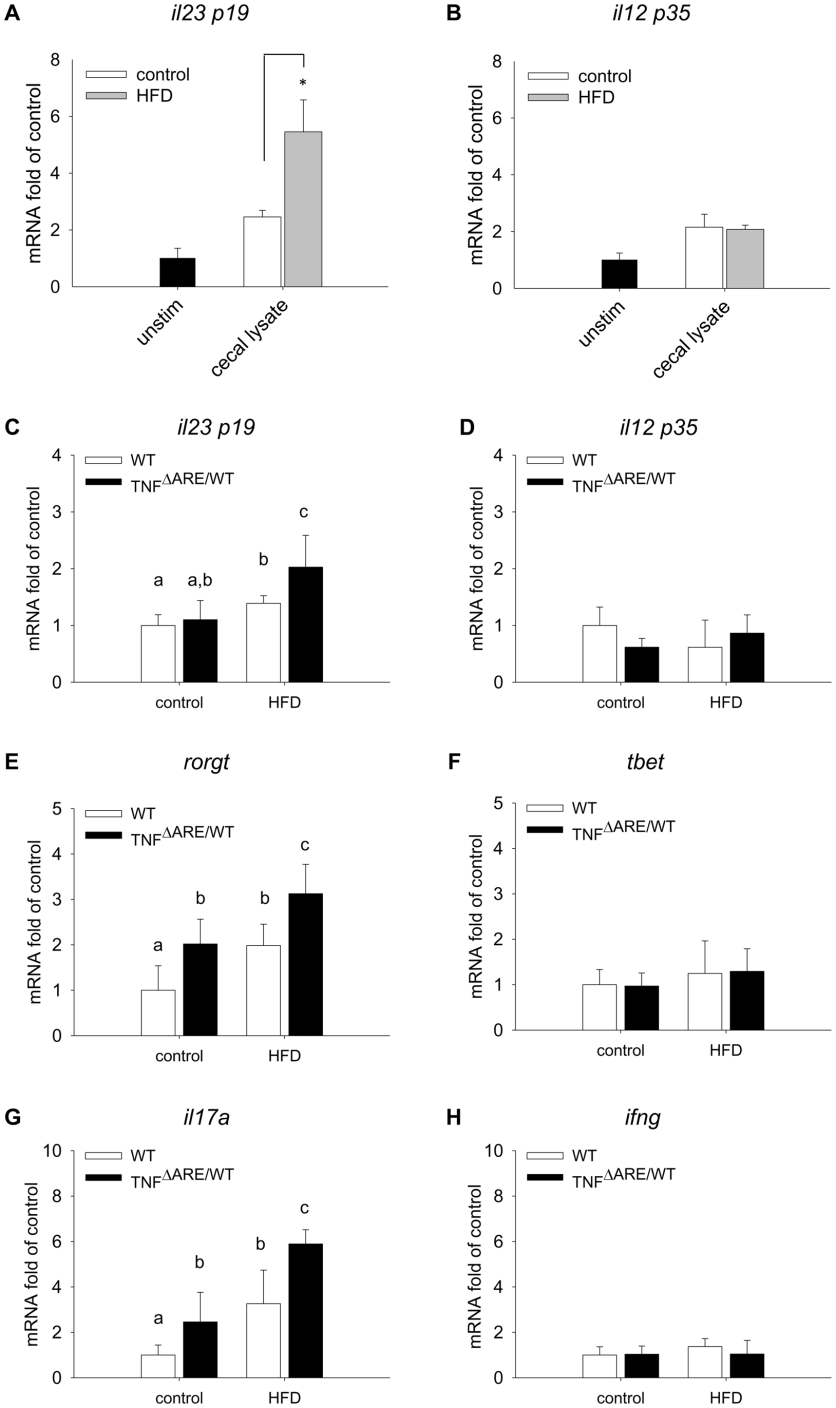
Diet-induced luminal changes are associated with Th17 biased immune response. *Il23p19* (A) and *il1235p35* (B) mRNA expression of BM-DCs was quantified by qPCR after 24 h stimulation with CM from Mode K cells treated with cecal lysate (50 µg protein/ml) of mice fed the different diets or unstimulated Mode K cells. Experiments were performed in triplicates. Expression of the cytokines *il23p19* (C) and *il12p35* (D), the transcription factors *rorgt* (E) and *tbet* (F), and the effector cytokines *il17a* (G) and *ifng* (H) were quantified in isolated LPLs using qPCR. mRNA expression was normalized to *gapdh* expression and expressed as –fold change relative to WT on control diet (n = 6 per group). Data sets with different superscript letters differ significantly from each other; *p<0.05 according to Student’s t-test.

To further evaluate the prospective effect of luminal factors on immune activation, we analyzed the effect of cecal lysates on epithelial cell-mediated activation of DCs. The activation status of BM-DCs, assessed as expression of co-stimulatory factor CD86 and antigen-presenting major histocompatibility complex MHCII (Figure S2), was not different between treatments with conditioned culture medium derived from cecal lysate-stimulated epithelial cell cultures. However, conditioned medium from Mode K cells stimulated with HFD–derived cecal lysate primed BM-DCs towards increased expression of Th17-conditioning *il23p19* (Figure 8A), but not toward Th1 conditioning *il12p35* (Figure 8B). Indeed, expression analysis of mouse LPLs revealed an induction of *il23p19* expression (Figure 8C), and no significant alterations in *il12p35* expression (Figure 8D) in HFD-fed TNF^ΔARE/WT^ mice. Similar to the *in vitro* results, significant up-regulation of the Th17-associated transcription factor *rorgt* (Figure 8E), but not the Th1-associated transcription factor *tbet* (Figure 8F) was observed in the LP compartment. Accordingly, *il17a* expression was increased (Figure 8G) and *ifng* expression remained unaltered (Figure 8H) in the ileal LP compartment.

## Discussion

We here show that a palm-oil based HFD accelerates the onset of small intestinal inflammation in TNF^ΔARE/WT^ mice, a model of CD-like ileitis, and additionally promotes expansion of inflammation into the proximal colon. HFD feeding does not result in the development of obesity or obesity-associated metabolic phenotypes such as glucose intolerance or changes in mesenteric fat tissue of TNF^ΔARE/WT^. We hypothesize that the observed aggravation of ileitis in the present study is independent of obesity, but is rather a consequence of altered luminal composition, increased intestinal permeability and so-called metabolic endotoxemia secondary to dietary lipids.

TNF^ΔARE/WT^ mice were protected from HFD-induced obesity and associated consequences such as increased cytokine and adipokine levels or impaired glucose tolerance. This is in line with our previous observations that this mouse model exhibits reduced body weight and adipocyte size compared to WT mice [13]. The intrinsic overexpression of TNF, shown here for the adipose tissue compartment, likely suppresses lipogenesis and promotes fat oxidation [20], [21], and accounts for this effect. Our finding that luminal factors and not the development of obesity and obesity-associated metabolic parameters are prerequisites for aggravation of IBD is supported by the results of a recent prospective cohort study reporting a lack of association of body mass index with IBD development [22]. In addition, recent evidence from the IL10^−/−^ colitis model demonstrated the importance of diet-induced alterations in the intestinal microbiota associated with the selection of the colitis-relevant pathobiont *Bilophila wadsworthia* [23].

Studies on the effect of HFD in the large intestine have previously shown a loss of colonic Occludin expression [24], [25]. In contrast, we here report a dramatic loss of Occludin in the distal ileum occuring upon HFD feeding independently of obesity and inflammation. Occludin seems to be one of the major targets for barrier disruption in the context of HFD feeding. Further studies by other groups have highlighted that increased permeability during diet-induced obesity is fat-driven and not obesity-driven, and probably related to bile acid composition [7], [9]. The impaired Occludin expression observed in the present study might contribute to an increased translocation of microbiota-derived endotoxin under HFD, often referred to as ‘metabolic endotoxemia’, and therefore trigger proinflammatory mechanisms leading to recruitment and activation of immune cells. While WT mice can obviously adjust to the disturbed barrier function and increase in endotoxin translocation without obvious loss of intestinal homeostasis, the intrinsic overexpression of TNF in the genetic mouse model leads to the exacerbated immune activation in response to the HFD challenge.

Epithelial cell signals are crucial for driving the recruitment and differentiation of DCs. We demonstrated that the expression and secretion of CCL20 and the subsequent recruitment of DCs is amplified *in vivo* upon HFD feeding. Using an *in vitro* model as an approach to understand underlying mechanisms allowed us to hypothesize that HFD-associated luminal factors stimulate epithelial signals to drive BM-DC recruitment and thereby may initiate inflammatory processes. While stimulation of the Mode K cell line with lysates of the different pure diets fail to mimic this effect, the lysed cecal content of HFD-fed mice are able to differentially promote CCL20 production. These findings emphasize the importance of luminal factors others than dietary lipids in promoting disease activity. IEC have been identified as the main producers of CCL20 in inflamed epithelium under different inflammatory conditions [26], and increased CCL20 expression is validated in colonic biopsies of UC and CD, but not in non-IBD colitis patients [27]–[29]. CCL20 expression is induced upon LPS or TNF/IL1β stimulation [26], suggesting a synergistic effect of TNF overproduction in the transgenic mouse model as well as luminal factors under HFD.

C-C chemokine receptor type (CCR) 6, the sole receptor known for CCL20, is expressed on immature DCs. Its expression is down-regulated upon activation in the LP, with concomitant initiation of CCR7 expression to allow migration to the mesenteric lymph node. Of note, CCR6 is implicated in the IBD pathogenesis based on genome-wide associations studies (GWAS) [30], and clinically most important, treatment with an anti-CCL20 neutralizing antibody significantly reduced the severity of TNBS-induced colitis in mice, with concomitant reduction of CCR6-bearing infiltrated LP T-cells [31]. CCR6 is also expressed on Th17 cells [32], and regulates Th17 cell migration to the gut [33]. Consistently, our results suggest that the expression of Th17-associated genes is upregulated in the lamina propria *in vivo*. In our *in vitro* model, the expression of Th17-directing *il23p19* is enhanced by epithelial signals under HFD-associated luminal conditions, promoting the generation of RORyt^+^ Th17 cells. It has been reported that obesity induced by HFD feeding promotes expansion of the Th17 cell lineage in mice [34]. Our present results indicate that these findings are related to alterations of luminal factors as well as epithelium-derived signals rather than the state of obesity.

In summary, this study shows that HFD feeding accelerates pathogenesis of CD-like ileitis in the TNF^ΔARE/WT^ mouse model. Based on the observations using the Mode K epithelial cell line and BM-DCs, we hypothesize that the disease activity-stimulating effects are attributed to alterations in the luminal compartment. Subsequent epithelial cell activation and DC-driven Th17 differentiation seem to be independent of obesity-related metabolic parameters but rather directly diet-induced effects.

## Supporting Information

Figure S1 **SWR/J mice do not develop HFD-induced obesity.** SWR/J mice were fed control diet or HFD from the age of 4 weeks until the age of 12 weeks. Body weight was assessed (A), and epididymal (B) and mesenteric (C) adipose tissue weights were determined. n = 6 per group; *p<0.05 according to Student’s t-test.
(TIF)

Figure S2 **Epithelial cell supernatants do not differentially stimulate CD86 or MHCII expression in BM-DCs.** Epithelial cell conditioned Mode K cells were stimulated with cecal lysates (50 µg protein/ml) of mice fed the different diets for 24 h. Media from unstimulated Mode K cells, or Mode K cells stimulated with CCL20 (200 pg/ml) were used as additional controls. The proportion of CD11c^+^ cells coexpressing CD86 (A) or MHCII (B) was assessed by cell cytometry after 24 h stimulation with the respective controls or Mode K cell conditioned media. Data sets with different superscript letters differ significantly from each other according to Student’s t-test with Bonferroni correction for multiple comparisons.
(TIF)

Table S1 **Composition of the diets.**
(DOC)

Table S2 **Most discriminating metabolites in plasma according to metabolite analysis using the Biocrates Life Sciences AbsoluteIDQ.**
(DOC)

Materials and Methods S1 (DOC)
