# Supplementary figures and images for: High Fat Diet Accelerates Pathogenesis of Murine Crohn’s Disease-Like Ileitis Independently of Obesity
Source: PLoS One. 2013 Aug 16;8(8):e71661. doi: 10.1371/journal.pone.0071661 (PMC3745443; doi:10.1371/journal.pone.0071661)

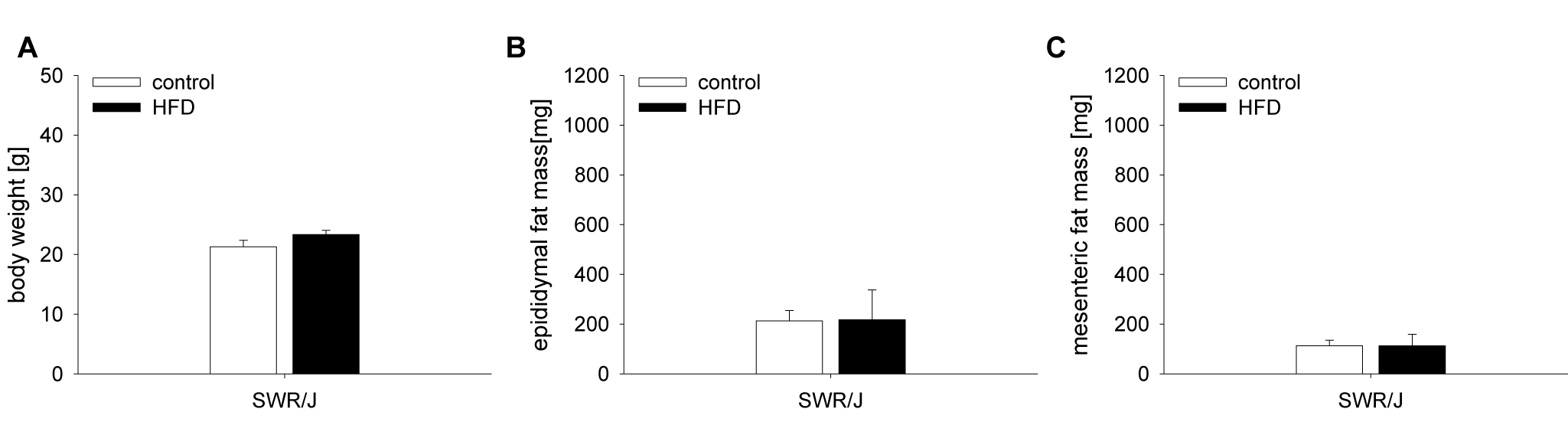

Supplement: Figure S1 — SWR/J mice do not develop HFD-induced obesity. SWR/J mice were fed control diet or HFD from the age of 4 weeks until the age of 12 weeks. Body weight was assessed (A), and epididymal (B) and mesenteric (C) adipose tissue weights were determined. n = 6 per group; *p<0.05 according to Student’s t-test. (TIF) [file pone.0071661.s001.tif]

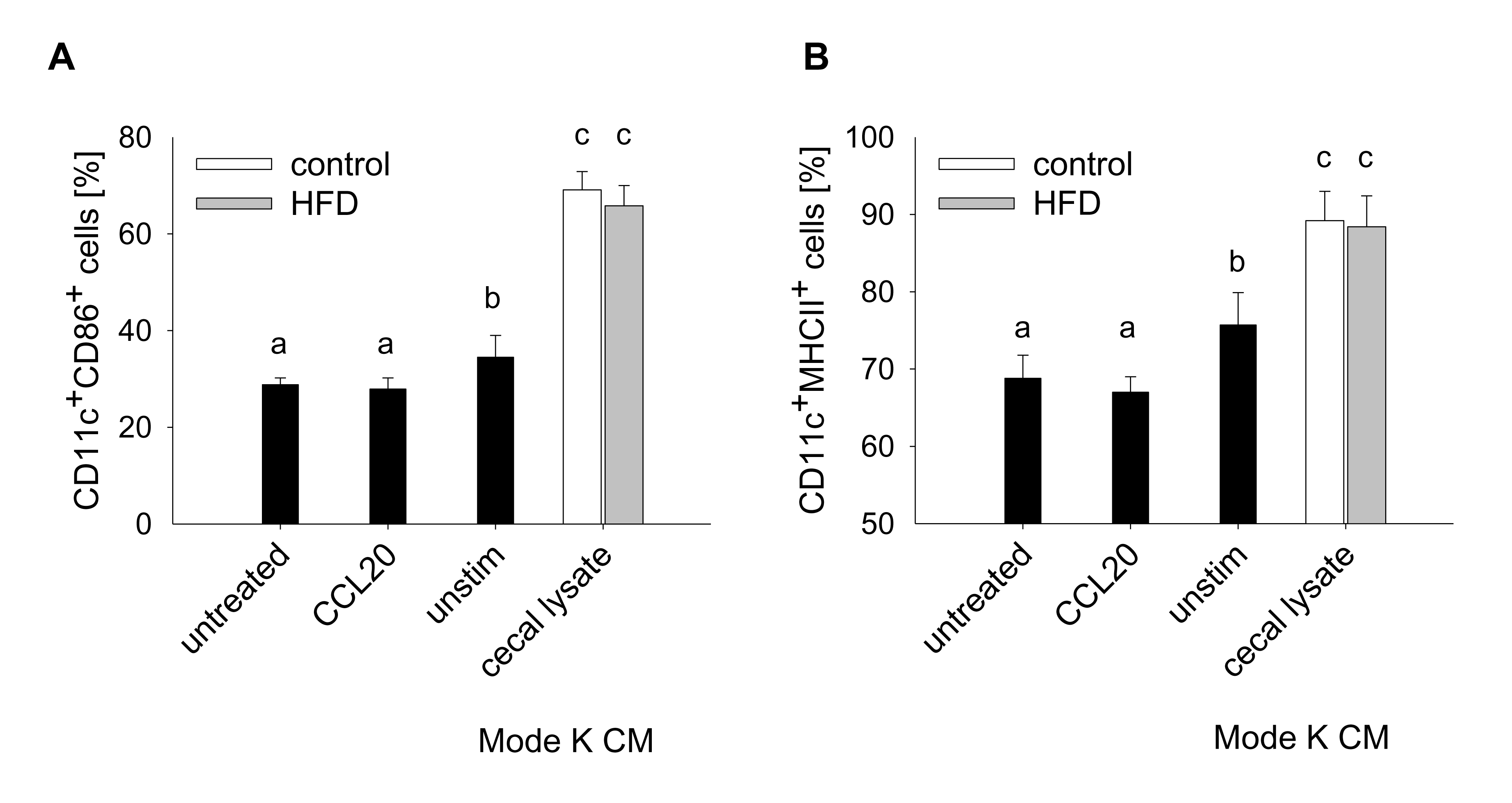

Supplement: Figure S2 — Epithelial cell supernatants do not differentially stimulate CD86 or MHCII expression in BM-DCs. Epithelial cell conditioned Mode K cells were stimulated with cecal lysates (50 µg protein/ml) of mice fed the different diets for 24 h. Media from unstimulated Mode K cells, or Mode K cells stimulated with CCL20 (200 pg/ml) were used as additional controls. The proportion of CD11c+ cells coexpressing CD86 (A) or MHCII (B) was assessed by cell cytometry after 24 h stimulation with the respective controls or Mode K cell conditioned media. Data sets with different superscript letters differ significantly from each other according to Student’s t-test with Bonferroni correction for multiple comparisons. (TIF) [file pone.0071661.s002.tif]
