# Supplementary material for: High Fat Diet Accelerates Pathogenesis of Murine Crohn’s Disease-Like Ileitis Independently of Obesity
Source: PLoS One. 2013 Aug 16;8(8):e71661. doi: 10.1371/journal.pone.0071661 (PMC3745443; doi:10.1371/journal.pone.0071661)
Supplement: Table S1 — Composition of the diets. (DOC) [file pone.0071661.s003.doc]

**Table S1. Composition of the diets.**

|  | **control diet (S575-E712)** | | **high-fat diet (S575-E702)** | |
| --- | --- | --- | --- | --- |
| Metabolizable energy [MJ/kg] | | 15.5 | 19.7 |  |
| Protein [kJ%] | | 23 | 18 |  |
| Fat [kJ%] | | 12 | 48 |  |
| Carbohydrates [kJ%] | | 65 | 34 |  |
| Casein [weight%] | | 24 | 24 |  |
| Corn starch [weight%] | | 47.8 | 27.8 |  |
| Soy oil [weight%] | | 5 | 5 |  |
| Palm oil [weight%] | | - | 20 |  |
| Cellulose [weight%] | | 5 | 5 |  |
| Maltodextrin [weight%] | | 5.6 | 5.6 |  |
| Sucrose [weight%] | | 5 | 5 |  |
| Vitamin mix [weight%] | | 1.2 | 1.2 |  |
| Mineral / trace element mix [weight%] | | 1.2 | 1.2 |  |
| Calcium [weight%] | | 0.92 | 0.92 |  |
| Phophate [weight%] | | 0.63 | 0.63 |  |
| Sodium [weight%] | | 0.19 | 0.19 |  |
| Magnesium [weight%] | | 0.21 | 0.21 |  |
| Lysine [weight%] | | 1.71 | 1.71 |  |
| Methionine [weight%] | | 0.75 | 0.75 |  |
| Methionine + cysteine [weight%] | | 1.04 | 1.04 |  |
| Threonine [weight%] | | 0.93 | 0.93 |  |
| C14:0 [weight%] * | | 0.03 | 0.24 |  |
| C16:0 [weight%] * | | 0.76 | 9.34 |  |
| C16:1 [weight%] * | | 0.01 | 0.04 |  |
| C18:0 [weight%] * | | 0.24 | 1.02 |  |
| C18:1 [weight%] * | | 1.32 | 8.57 |  |
| C18:2 [weight%] * | | 2.78 | 4.28 |  |
| C18:3 [weight%] * | | 0.02 | 0.38 |  |
| C20:0 [weight%] | | 0.03 | 0.13 |  |
| C20:1 [weight%] * | | 0.01 | 0.04 |  |
| C22:0 [weight%] * | | 0.03 | 0.04 |  |

* analyzed by Bioanalytik Weihenstephan (ZIEL, Technische Universität München); see Supporting Methods S1
