# Supplementary material for: High Fat Diet Accelerates Pathogenesis of Murine Crohn’s Disease-Like Ileitis Independently of Obesity
Source: PLoS One. 2013 Aug 16;8(8):e71661. doi: 10.1371/journal.pone.0071661 (PMC3745443; doi:10.1371/journal.pone.0071661)
Supplement: Table S2 — Most discriminating metabolites in plasma according to metabolite analysis using the Biocrates Life Sciences AbsoluteIDQ. (DOC) [file pone.0071661.s004.doc]

**Table S2. Most discriminating metabolites in plasma according to metabolite analysis using the Biocrates Life Sciences AbsoluteIDQ.**

| **metabolite** | **-fold regulation by HFD** |
| --- | --- |
| C16 | 2.7±0.8 *** |
| C18 | 2.1±0.7 *** |
| C18:1 | 2.6±1.0 *** |
| PC aa C36:1 | 2.0±0.6 *** |
| lysoPC a C20:3 | 1.4±0.3 *** |
| lysoPC a C16:0 | 1.6±0.3 *** |
| PC aa C34:1 | 1.9±0.7 *** |
| lysoPC a C18:1 | 1.7±0.4 *** |

C: acylcarnitine, PCaa: choline glycerophospholipid, lysoPC: lysophosphatidylcholine
