# Supplementary material for: High Fat Diet Accelerates Pathogenesis of Murine Crohn’s Disease-Like Ileitis Independently of Obesity
Source: PLoS One. 2013 Aug 16;8(8):e71661. doi: 10.1371/journal.pone.0071661 (PMC3745443; doi:10.1371/journal.pone.0071661)
Supplement: Materials and Methods S1 — (DOC) [file pone.0071661.s005.doc]

**Methods S1**

**Analysis of energy content in feces**

Fecal samples were collected from individual mice at indicated timepoints, dried at 60°C for 3 days, and stored airtight until being assessed. Fecal pellets were manually ground and pressed into pellets and the gross energy content was determined using a 6300 Calorimeter (Parr Instrument Company, Moline, IL, USA). Fecal composition was analyzed by diffuse reflection using Fourier transform infrared spectrometry (FT-IR) on a HTS-XT spectrophotometer (Bruker Optics, Ettlingen, Germany). Spectra were obtained in the range of 6000cm-1 to 600cm-1. Data were analyzed and calculation of first deviation, vector normalization and cluster analysis according to Ward’s algorithm were performed using the OPUS Software version 6.5 (Bruker).

**Metabolite analyses in plasma and ileal tissue**

For targeted LC-MS analysis, a 1 cm-long sample piece of the distal ileum was excised and flushed with 1 ml phosphate buffered saline using a sterile syringe and stored at -80 °C. The Biocrates Life Sciences AbsoluteIDQ kit, originally designed for plasma samples, was adapted as previously published to be used for intestinal tissue. The tissue was homogenized in 300 μl EDTA (ethylenediaminetetraacetic acid; 0.292 mg/ml PBS) and 10 μl BHT-buffer (butylated hydroxytoluene; 79.2 mg/ml PBS) using the FastPrep 24 system (MP Biomedicals LLC, Illkirch, France). Afterward, well plate preparation and sample application and extraction were carried out according to the manufacturer’s instructions. A final volume of 10 μl of tissue homogenate was loaded onto the provided 96-well plate. Liquid chromatography was performed using a Dionex Ultimate 3000 ultra-high pressure liquid chromatography system (Dionex AG, Olten, Switzerland) coupled to a 3200 Q TRAP mass spectrometer (AB Sciex; Foster City, CA, USA) fitted with a TurboV ion source operating in electrospray ionization (ESI) mode. Sample extracts (20 μl) were injected two times (in positive and negative ESI modes) via direct infusion using a gradient flow rate of 0-2.4 min: 30 μl/min, 2.4-2.8 min: 200 μl/min, 2.9-3 min: 30 μl/min. MS source parameters were set at: esolvation temperature: 200 °C, high voltage: -4500 V (ESI -), 5500 V (ESI +), curtain and nebulizer gases: nitrogen; 20, 40, and 50 psi; respectively, nitrogen collision gas pressure: 5 mTorr. MS/MS acquisition was realized in scheduled reaction monitoring mode with optimized declustering potential values for the 163 metabolites screened in the assay. Raw data files (Analyst software, version 1.5.1; AB Sciex, Foster City, CA, USA) were imported into the provided analysis software MetIQ to calculate metabolite concentrations. The list of all detectable metabolites is available from Biocrates Life Sciences, Austria (http://biocrates.com). To identify the metabolites separating sample groups the supervised pattern recognition methods Partial Least Squares (PLS) and Orthogonal PLS (O-PLS) discriminant analysis (PLS-DA and O-PLS-DA) were applied. All models were generated using one predictive and one orthogonal component to discriminate between two groups of animals. The model robustness was assessed using the standard 7-fold cross validation method (repeatedly leaving out a seventh of the samples and predicting them back into the model). Also R²X and R²Y values were computed to show how much of the variation in the data set X (metabolite data) and in the data set Y (group information) was explained by the model. The validity of the model against overfitting was monitored by computing the cross-validation parameter Q² which represents the predictability of the models and relates to the statistical significance. Negative or very low values of the Q² indicated that no statistically significant differences were observed. In addition, Mann-Whitney U tests were performed on variables to assess significant differences of metabolite concentrations, respectively.

**Analysis of fatty acid profile of the diets**

To ensure that the mice were actually provided with the nutrients as stated by the manufacturer, we had the macronutrients as well as fatty acid concentration in the lots used in these feeding studies re-assessed by Bioanalytik Weihenstephan (ZIEL, Technische Universität München). Analyses was performed according to the guidelines published in . Fatty acid profiles of the diets were largely comparable to the manufacturer’s statements, but additional fatty acids were investigated (see Supporting Table S1).

**Polyethylene glycol permeability assay**

Polyethylene glycols (PEG) (Merck, Darmstadt, Germany) of the average molecular sizes 1500 and 3000 Da were applied solubilized in PBS to 12-week-old mice, at final amount of 1.5 mg and 5 mg per mouse respectively, and urine was collected for 24 h. Urine samples were diluted to a theoretical osmolarity of 500 mosmol in acetonitrile water (1:9) for subsequent analysis via LC/MS. Samples were analyzed in duplicates. MS spectra were obtained from a hybrid triple-quadrupole/linear ion trap mass spectrometer (API 4000 QTrap; Applied Biosystems Inc., Foster City, CA, USA). The ion source (Turbo Ion Spray) was operated in the positive electrospray ionization (ESI) mode. Settings were as follows: curtain gas (CUR): 20 psi, (IS): 5500 V, temperature (TEM): 450 °C, ion source gas 1 (GS1): 45 psi, ion source gas 2 (GS2): 55 psi. For LC-MS measurements, the mass spectrometer was operated in the SIM (single ion monitoring) mode. Data acquisition was carried out using Analyst 1.5 software (Applied Biosystems). HPLC separation prior to mass spectrometric detection was performed on a Shimadzu LC-20A prominence HPLC system (Shimadzu, Kyoto, Japan). As stationary phase a 150 mm x 3.0 mm i.d. 3 μm Gemini-NX (Phenomenex, Aschaffenburg, Germany) was used. The mobile phase was mixed water (A) and acetonitrile (B) following a linear binary gradient as follows: Initial conditions were 15% B and 85% A. The content of solvent B was linearly raised during the next 10 min to obtain 30% B and 70% A. During the next 15 min the content of B was linearly raised to 40% and during the next minute to 50%. These conditions were held for 3 min, before the content of solvent B reached initial conditions after 1 min. Injection volume was 10 μl, flow rate was 0.2 ml/min and equilibration time was between two runs 10 min. PEG were calculated as sum of the sodium adducts.

**Cecal content preparations**

Cecal lysates were prepared from cecal contents of 12-week-old WT C57BL/6 mice having received the different experimental diet for 8 weeks. The preparation was adapted from a previously described protocol. Briefly, the cecum was dissected, the cecal content collected and snap-frozen. It was stored at -80°C until usage, then diluted in sterile PBS (1:1) and vortexed thoroughly. After centrifugation at 8000 g for 5 min, cecal water was collected. For generation of whole cecal lysates, cecal suspensions (0.5 ml) were mixed with PBS and one volume of 0.1 mm glass beads (Roth, Karlsruhe, Germany) and disrupted in a bead-beater (FastPrep-24, MP Biomedicals LLC) at 5000 Hz for 0.5 min, at total six cycles interrupted by 2 min on ice each. The glass beads and unlysed cells were removed by centrifugation at 350 g for 5 min. Supernatants were collected and centrifuged at 8000 g, 5 min. After centrifugation, the supernatant was filtered through a prewashed 0.45 μm filter. The protein concentration in each fraction was assessed using Bradford method assay according to the manufacture’s protocol (Roti-Quant, Roth, Karlsruhe, Germany). Cecal lysates were stored at −20°C.

1. Baur P, Martin FP, Gruber L, Bosco N, Brahmbhatt V, et al. (2011) Metabolic phenotyping of the Crohn's disease-like IBD etiopathology in the TNF(DeltaARE/WT) mouse model. J Proteome Res 10: 5523-5535.

2. Thalmann A (1976 ) Handbuch der Landwirtschaftlichen Versuchs- und Untersuchungsmethodik, Methodenbuch des Verbandes Deutscher Landwirtschaftlicher Untersuchungs-. und Forschungsanstalten e.V. Darmstadt: VDLUFA Verlag.

3. Albright CA, Sartor RB, Tonkonogy SL (2009) Endogenous antigen presenting cell-derived IL-10 inhibits T lymphocyte responses to commensal enteric bacteria. Immunol Lett 123: 77-87.
